# Impact of Fatty Acid Carbon Chain Length and Protein Composition on Physicochemical and Digestive Properties of MFGM Contained Emulsions

**DOI:** 10.1002/fsn3.70220

**Published:** 2025-07-07

**Authors:** Yanchen Liu, Ning Wang, Yunpeng Xu, Zihao Guo, Guangqing Mu, Xuemei Zhu

**Affiliations:** ^1^ School of Food Science and Technology Dalian Polytechnic University Dalian P. R. China

**Keywords:** emulsifier content, fat digestibility, MFGM emulsions, physicochemical properties, triacylglyceride carbon chain length

## Abstract

Improving fat digestibility is crucial for improving fatty acid intake in populations with weak digestive absorption capacities. Currently, research often analyzes the impact of triacylglyceride carbon chain length and emulsifier content on lipid digestion and absorption individually, overlooking their combined effects. This study aims to provide a reference for addressing the differences in fat digestibility between infant formula and breast milk by investigating the combined influence of these two factors on the physicochemical properties of droplet hydrolysis. The findings reveal significant differences in the interface substances and properties of lipid droplets composed of fatty acids with various carbon chain lengths. Emulsions prepared by OPO (long‐chain fatty acids) exhibited the largest average particle size and greater stability compared to those with medium‐chain fat (coconut oil) and ultra‐long‐chain fat (DHA algae oil). In addition, the protein composition and content on the surface of lipid droplets influenced the physical and chemical properties of emulsions. When whey protein and casein were added to a 6:4 ratio, the effect of fatty acid carbon chain length on the average particle and the emulsification characteristics of the lipid droplet interface were significantly reduced. Furthermore, emulsions composed of fat globules with a core of short‐chain fatty acid or low surface protein content had higher lipolysis rates. These findings provide valuable insights for improving nutrient bioavailability in future food product developments, especially in enhancing infant nutrition.

## Graphical abstract

Emulsions prepared by OPO (long‐chain fatty acids) exhibited the largest average particle size and greater stability compared to those with medium‐chain fat (coconut oil) and ultra‐long‐chain fat (DHA algae oil). When whey protein and casein were added in a 6:4 ratio, the effect of fatty acid carbon chain length on the average particle and the emulsification characteristics of the lipid droplet interface were significantly reduced. Emulsions composed of fat globules with a core of short‐chain fatty acids or low surface protein content had higher lipolysis rates.

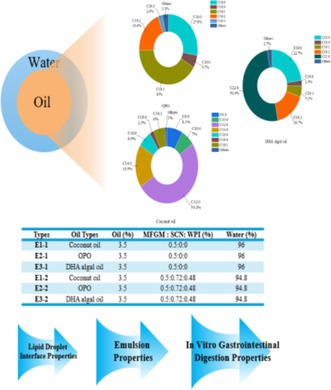

## 1 Introduction

The structure of the fat globule interface plays a critical role in the hydrolysis of fat droplets by lipases, subsequently influencing the digestion and absorption of fat. Research indicates that the composition of the fat droplet surface exerts a greater influence on digestion than the size of the droplets (Luo et al. 2019). In breast milk, the surface of fat globules is composed of a tri‐layer of phospholipid molecules embedded with proteins, glycoproteins, lipoproteins, enzymes, and cholesterol, whereas in infant formula, the surface of fat globules primarily consists of whey protein and casein, highlighting significant differences in the surface properties of fat globules between the two (Pan et al. 2022; Yuan et al. 2020). Consequently, commercial milk fat globule membranes (MFGM) and polar lipids are often added to infant formula emulsions to mimic the interface of breast milk fat globules better. The addition of MFGM components not only reduces the metabolic differences between breastfed and formula‐fed infants but may also affect the physicochemical properties of the emulsion and the release rate of fatty acids (Lee et al. 2021). Studies exploring emulsions prepared by blending MFGM‐enriched whey protein concentrate with mixed vegetable oils have shown that the rate of fat breakdown and the release rate of fatty acids are significantly higher in droplets containing MFGM than in those enclosed solely by protein (Sun et al. 2023a). Adjusting the interface structure of fat globules containing mixed oils by combining phospholipids from different sources with MFGM may be an effective strategy to improve the stability of simulated human milk fat globule emulsions and lipid digestion (Ma et al. 2024).

The core of fat globules, triglycerides, may also influence the bioavailability of fat globules through their interaction with lipase and their effect on fat droplets. In natural fat globules, those with smaller diameters are rich in medium‐chain triglycerides, whereas larger globules contain triglycerides with longer carbon chains (Timmen and Patton 1988). Different‐sized fat globules have varying contact areas with lipase, thereby limiting the rate of hydrolysis. In infant formula, coconut oil, or milk fat are typically added to increase the content of medium‐chain fatty acids. Research indicates that triglycerides containing short or medium‐chain fatty acids are hydrolyzed more rapidly by gastrointestinal enzymes, without the need for bile salts, and their hydrolysates are more readily absorbed (Mazzocchi et al. 2018). And after digestion, medium‐chain and long‐chain fatty acids can enter mitochondria for β‐oxidation without the assistance of carnitine palmitoyltransferase (Schonfeld and Wojtczak 2016).

Furthermore, emulsifiers attached to the surface of fat globules can also affect the size and surface properties of the fat globules. Whey protein isolates (WPI) in emulsions are usually dispersed in the aqueous phase in a dissolved state, while casein typically exists in the form of micelles in the aqueous phase. Negatively charged casein micelles interact with polar lipids in the liquid‐disordered phases region of the fat globule interface, and the significant intermolecular forces between polar lipids facilitate protein adsorption on the surface of the fat globule (Obeid et al. 2019). Proteins adsorbed at the oil–water interface may affect the stability, particle size, surface charge of fat droplets, and rheological properties of the emulsion. Studies have shown that when the ratio of sunflower phospholipids to proteins is adjusted from 1:8 to 1:1, the size of the emulsion droplets significantly decreases. Additionally, the reduction in protein content decreases the surface tension and viscosity of the emulsion, further enhancing its stability (Chen et al. 2020).

Currently, most studies focus on investigating the effects of either the surface structure and composition of fat globules or the emulsifier content in emulsions on the physicochemical properties and digestion characteristics of fat droplets, overlooking the combined influence of triglycerides, the core of fat globules, and emulsifiers in emulsions. Conventional infant formulae typically comprise a blend of vegetable oils or are supplemented with bovine milk fat to compensate for the differences in fatty acid composition between single oil fats and breast milk fats, meeting the nutritional needs for infant growth and development. Additionally, they are fortified with DHA and ARA to further optimize the fatty acid composition in infant formulae (Hageman et al. 2019). The varying carbon chain lengths of triglycerides exert different effects on lipid digestion and absorption, especially in populations with weak digestive capabilities. Therefore, it is essential to investigate the combined influence of different carbon chain length triglycerides and emulsifier concentrations on the physicochemical properties and digestive characteristics of emulsions.

Therefore, in this study, emulsions were prepared using coconut oil (containing medium‐chain polyunsaturated fatty acids), 1,3‐dioleoyl‐2‐palmitoylglycerol (OPO) (containing medium‐to‐long‐chain fatty acids), and DHA algae oil (containing long‐chain polyunsaturated fatty acids), along with commercialized MFGM, WPI, and sodium caseinate (SCN) (Table 1). We explored the combined effects of triglyceride carbon chain length and emulsifier content on the physicochemical properties and digestion characteristics of emulsions. This aims to bridge the gap between triglyceride composition, emulsifier content, and their joint effect on lipid digestion in emulsions, offering new insights for the development of infant formula or improved fat‐based food formulations.

**TABLE 1:** Formulation of the emulsions.

Types	Oil types	Oil (%)	MFGM:SCN:WPI (%)	Water (%)
E1‐1	Coconut oil	3.5	0.5:0:0	96
E2‐1	OPO	3.5	0.5:0:0	96
E3‐1	DHA algal oil	3.5	0.5:0:0	96
E1‐2	Coconut oil	3.5	0.5:0.72:0.48	94.8
E2‐2	OPO	3.5	0.5:0.72:0.48	94.8
E3‐2	DHA algal oil	3.5	0.5:0.72:0.48	94.8

## 2 Materials and Methods

### 2.1 Materials and Reagents

OPO was sourced from Jinhai Food Industry Co. Ltd. (Hebei, China), while DHA algal oil (Docosahexaenoic Acid content 42.9%) was procured from Jiangsu Grand Xianle Pharmaceutical Co. Ltd. (Jiangsu, China). Coconut oil and SCN (protein 90%–100%) were obtained from Shanghai Macklin Biochemical Technology Co. Ltd. (Shanghai, China), and WPI (protein 80%) was acquired from Shanghai Yuanye Bio‐Technology Co. Ltd. (Shanghai, China). Milk fat Globule Membrane Enriched Whey Protein Concentrate (protein 70%, phospholipid 6.5%, fat 15%) was purchased from Galaxy Weiye Import and Export Co. Ltd. (Tianjin, China).

Trypsin was purchased from Macklin Biochemical Technology Co. Ltd. (Shanghai, China). Other enzymes used in in vitro digestion were obtained from Sigma‐Aldrich (Shanghai, China). Bile Salt was purchased from Shanghai Yuanye Bio‐Technology Co. Ltd. (Shanghai, China). All other chemicals were of analytical grade and were procured from Dalian Bonuo Biochemical Reagent Factory (Liaoning, China).

### 2.2 Fatty Acid Methyl Esters

Fatty acid methyl esters were prepared following the method described by Liu (1994), with slight modifications. The bands corresponding to free fatty acids (FFA) and 2‐MAG were scraped off and dissolved in 5 mL of hexane. A 0.5 mL aliquot was then filtered and dried. Next, 2 mL of NaOH‐methanol solution was added, and the mixture was subjected to condensation reflux at 80°C for 5 min. Afterward, 2 mL of boron trifluoride‐methanol was introduced through the top of the condenser, and the mixture was heated in a water bath for an additional 2 min. Once removed, the solution was cooled to room temperature, followed by the addition of 1.5 mL of hexane. The mixture was vortexed for 200 s and allowed to separate. The upper layer was collected, passed through anhydrous sodium sulfate, and stored at 4°C for 30 min. Finally, the solution was filtered through a 0.22‐μm membrane into a sample vial for further analysis.

### 2.3 Oil Fatty Acid Composition Analysis

Following the GB5009.168–2016 standard, the total fatty acid composition of coconut oil, OPO, and DHA algae oil was analyzed using gas chromatography after methylation. Fatty acid analysis was performed using an Agilent 7890B gas chromatograph (Agilent Technologies, California, USA) equipped with a hydrogen flame ionization detector and a CP‐Sil88 fused silica capillary column (100 m x 0.25 mm). High‐purity hydrogen was used as the carrier gas at a flow rate of 30 mL/min, with the column temperature maintained at 225°C. A 2 μL sample was injected, and the detector temperature was set to 250°C. The temperature program was as follows: the initial temperature of 45°C was held for 4 min, then increased at a rate of 10°C per minute to 175°C, held for 27 min, and finally raised to 225°C, holding for 35 min. The relative concentration (RC) of fatty acids was determined using the area normalization method, and the relative content (%) of fatty acids in each sample was analyzed.

### 2.4 Emulsion Preparation

In accordance with the methodology described by Zhu et al. (2021), six emulsions were prepared following the formulations in Table 1 (Pan et al. 2024). Initially, the emulsifiers: MFGM or a combination of MFGM, WPI, and SCN were dissolved in deionized water and stirred overnight at 25°C using a magnetic stirrer (IKA‐Werke GmbH & Co. KG, Germany) to ensure complete hydration. Subsequently, coconut oil, OPO, and algal oil were individually incorporated into the protein solution and stirred at 500 rpm for 1 h at 50°C. The resulting aqueous and oil phases were then subjected to mixing and shear at 25°C for 2 min at 12000 rpm using a disperser (IKA‐Werke GmbH & Co. KG, Germany) to obtain primary emulsions. Further homogenization of the primary emulsion was conducted using a high‐pressure homogenizer (ATS Engineering Ltd., Canada) at 30 MPa pressure for 3 cycles.

### 2.5 Structural Characterization

#### 2.5.1 Particle Size and Zeta Potential

Following the method described by Li et al. (2020), the volume mean diameter (D_4,3_), volume particle volume size distribution, and ζ‐potential of the emulsion were determined using a laser particle size analyzer (Malvern Instruments Ltd., Britain). All measurements were conducted at room temperature. To minimize multiple scattering effects and accommodate the instrument's sensitivity, the emulsion sample was diluted 500‐fold with water and transferred into the cuvette for analysis.

### 2.6 Physical Characteristics

#### 2.6.1 Interfacial Protein Content

Interface proteins were extracted following the methodology described by Pan et al. (2022). Initially, 30 mL of the emulsion was centrifuged at 4°C for 30 min at 10,000 g. Subsequently, the lower liquid layer was carefully aspirated, while the cream phase (upper layer) was stored overnight at −20°C. The following day, the stored cream phase was thawed in a 45°C water bath and then subjected to centrifugation at 3500 g for 10 min. After removing the upper oil phase, the lower layer was mixed with chloroform‐methanol (2:1, v/v), and centrifuged at 3500 g for 10 min again. The intermediate layer was where interface proteins were located. After removing the upper and lower layers, interface proteins were solubilized in 5 mL of PBS solution, whose content was determined using a BCA assay kit (Beijing Solarbio Science & Technology Co. Ltd., China).

#### 2.6.2 Emulsifying Index

The determination of emulsifying activity index (EAI) and emulsion stability index (ESI) followed the methodology proposed by Liu et al. (2023) with appropriate adjustments. Samples were diluted to 40 mL using a 0.1% SDS solution. The absorbance of the emulsions at a wavelength of 500 nm and at room temperature was measured using a UV–visible spectrophotometer (Beijing Puxi General Instrument Co. Ltd., China) at 0 and 30 min. The EAI and ESI values were calculated using the following equations, with the SDS solution serving as the blank sample:

$$\mathrm{EAI}\;\left(\frac{m^{2}}{g}\right)=2\times T\times \frac{A_{0}\times N}{\rho \times 10000\times \psi }$$

$$\mathrm{ESI}\;\left(\min\right)=\frac{A_{0}}{A_{0}-A_{30}}\times 30$$

Where: T = 2.303; *N* = dilution factor; ρ = protein concentration in the aqueous protein solution before emulsion preparation, in g/mL; ψ = oil phase volume fraction in the emulsion (3.5); A_0_ = absorbance at 0 min; A_30_ = absorbance at 30 min.

#### 2.6.3 Rheological Properties Evaluation

The rheological behavior of the emulsion was characterized according to the methodology developed by Wang et al. (2017). It was performed using a KINEXUS rotational rheometer (Malvern Instruments Ltd., Britain) equipped with a P40 parallel plate geometry (40 mm in diameter), maintaining a gap of 1 mm between the plate and the sample surface. The evaluation was conducted under a frequency sweep mode, spanning from 0.1 Hz to 10 Hz, at a constant strain of 1% to ensure the measurements were within the linear viscoelastic region. This setup was utilized to ascertain the variations in the storage modulus (G') and loss modulus (G") across the specified frequency range.

### 2.7 In Vitro Digestion Simulation

The in vitro digestion process mimicked full‐term infant gastric and intestinal phases, following Menard et al. (2018) with modifications. Enzymatic activities were measured: pepsin (268 U/mL), gastric lipase (19 U/mL), trypsin (16 U/mL), intestinal lipase (90 U/mL), and bile salts (3.1 mmol/mL). In gastric digestion, simulated gastric fluid (SGF) preparation included 94 mM NaCl and 13 mM KCl (Sun et al. 2023b). Then 40 mL emulsion was mixed with 15 mL SGF and 5 mL SGF, including pepsin (600 U/mg) and lipase sourced from Rhizopus oryzae (30 U/mg). Digestion lasted 60 min with pH control at 5.3. Samples were taken at 0 and 60 min (G0 and G60). After the end of gastric digestion by adjusting the pH to 7, 25 mL of simulated intestinal fluid (SIF) (164 mM NaCl, 119 mM CaCl2, and 10 mM KCl) and 5 mL of SIF, including bile salts (0.5 mmol/g), pancreatic lipase (30,000 U/mg), and trypsin (250 U/mg) were added to the solution. The process lasted 180 min with pH control at 6.6. Samples were collected at 60, 120, and 180 min (I60, I120, and I180). Enzyme activity was stopped by heating samples to 95°C for 5 min.

#### 2.7.1 FFA Release Rate

FFA release was calculated from the consumption of 0.1 M NaOH during gastrointestinal digestion by using a T5 automatic potentiometric titrator (IKA‐Werke GmbH & Co. KG, Germany) (Tangsrianugul et al. 2015). The formula applied for the calculation is presented below:

$$\mathrm{FFA}\;\left(\%\right)=\frac{V\times C\times M}{2m}\times 100$$

Here, M represents the average molecular weight of the oil (g/mol), V denotes the volume of NaOH (mL) consumed throughout the gastrointestinal digestion, C signifies the molarity of the NaOH solution, and m is the mass of the oil present in the digestive liquid.

#### 2.7.2 Protein Digestibility Condition

The protein digestibility of six emulsions during the digestion process was determined with slight modifications to the method outlined by de Morais et al. (2020). Five milliliters of digested samples were treated with an equal volume of 24% trichloroacetic acid solution to precipitate proteins. Following centrifugation at 5000 rpm for 30 min, the supernatant was collected. The amino acid release rate of the samples at different time points in vitro digestion was determined by an automatic Kjeldahl nitrogen analyzer (Hanon Technologies Co. Ltd., China). Amino acids are compounds composed of amino groups and carboxyl groups, with the amino group containing nitrogen. Therefore, the nitrogen content measured in the supernatant can indirectly reflect the amount of free amino acids generated from protein digestion.

### 2.8 Statistical Analysis

Data presented in the figures are reported as means ± standard deviations. Differences among groups were evaluated using one‐way ANOVA, with post hoc comparisons conducted via LSD and Duncan's multiple range test, utilizing SPSS Statistics version 19 (SPSS Inc., USA). A threshold for statistical significance was established at *p* < 0.05. All analyses were carried out in triplicate to confirm the reliability and consistency of the conclusions.

## 3 Results and Discussion

### 3.1 Fatty Acid Composition of the Three Oils

**FIGURE 1:**
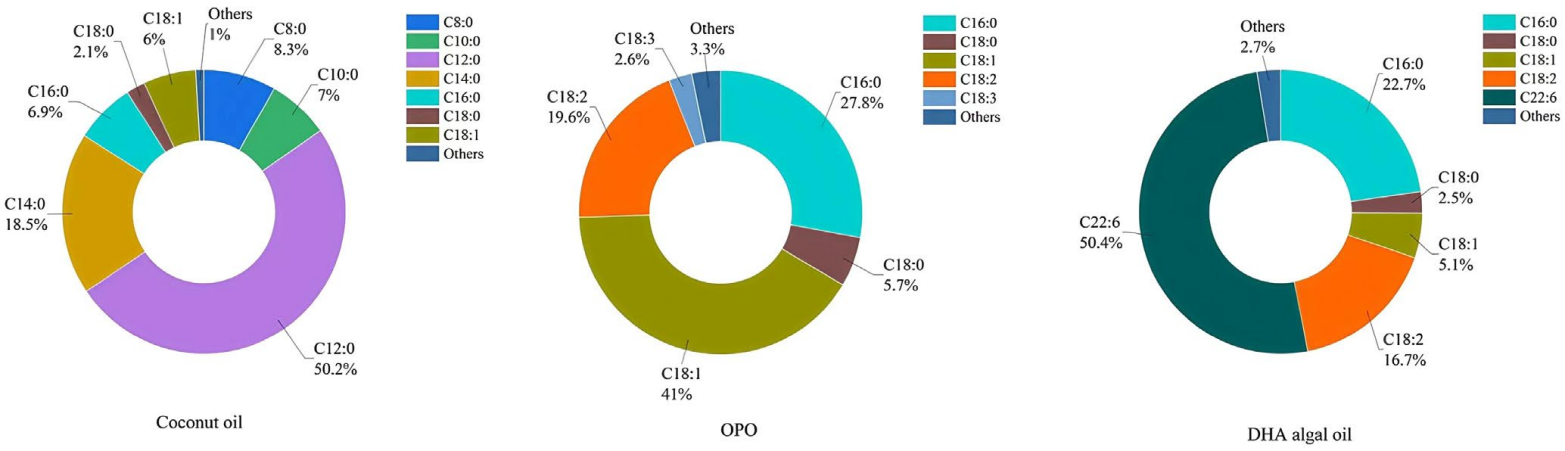
The main fatty acid compositions of the three oils.

By analyzing the composition of fats and oils, the differences in fatty acid composition among various oils can be precisely identified. There were significant differences in the carbon chain lengths of fatty acids in coconut oil, OPO, and DHA algal oil (Figure 1). In coconut oil, the fatty acid carbon chain lengths range mainly from 8 to 16. The most abundant fatty acids are lauric acid (C12), constituting 50.2% of the total fatty acids, and myristic acid (C14), making up 18.5%. Other notable fatty acids include caprylic acid (C8), capric acid (C10), and palmitic acid (C16). Saturated fatty acids account for over 90% of the total fatty acids in coconut oil. In OPO oil, the fatty acid carbon chain lengths primarily range from 16 to 18, with 18‐carbon fatty acids comprising 68.9% of the total. The content of saturated fatty acids is below 37%. DHA algae oil contains more than 50% docosahexaenoic acid (DHA, C22:6). Additionally, fatty acids with 16–18 carbon atoms constitute 47% of the total content, and saturated fatty acids do not exceed 30% of the total fatty acids. Due to the differences in fatty acid carbon chain lengths and the degree of unsaturation, these three oils represent distinct types of oils with different triglyceride spatial structures (Jensen 2002).

### 3.2 Initial Emulsions: Physicochemical Properties

#### 3.2.1 Lipid Droplet Surface Protein Enrichment Content and Zeta‐Potential

**FIGURE 2:**
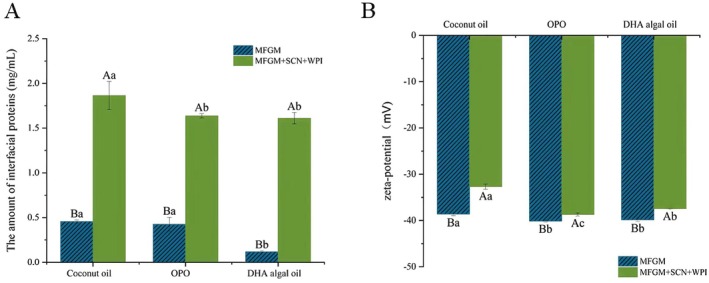
Effects of fatty acid carbon chain length and protein content in emulsions on surface protein enrichment content and zeta‐potential. The different lowercase letters indicate significant differences between the same emulsifiers in different oil phases (*p* < 0.05), while uppercase letters indicate significant differences between the same oil phases in different emulsifiers (*p* < 0.05).

The adsorption capacity of proteins is influenced by factors such as protein folding degree, stability, size, and hydrophobic residue distribution. Moreover, the polarity of lipids also dictates the protein adsorption kinetics (Bergfreund et al. 2021). The composition of the droplet surface correlates with the types of triglycerides and emulsifier systems. The protein content at the oil–water interface can reflect the degree of protein enrichment on the droplet surface (Figure 2A). In systems with only added MFGM, the protein enrichment content on the surface of droplets prepared from DHA algae oil was significantly lower than the other two emulsions. Upon additional supplementation of WPI and SCN to the system, no significant difference was observed in the protein content on the surfaces of DHA algae oil and OPO droplets, while the protein content on the surface of coconut oil droplets was significantly higher than these two oils. This phenomenon was attributed to the effect of the carbon chain length and spatial curvature of fatty acids in DHA algae oil on the distribution of phospholipids on the fat globule surface, thereby inhibiting the interaction between phospholipids and proteins on the droplet surface (Wang et al. 2023). Additionally, in systems with both emulsifiers, only a fraction of proteins is enriched on the droplet surface. This was due to temperature‐induced denaturation of proteins during preparation, leading to aggregation or complexation with lipoproteins, thereby dispersing in the aqueous phase, which impacted the degree of digestion of triglycerides and proteins further.

Furthermore, the protein enrichment on the surface of fat droplets also influences the charge density at the oil–water interface. As shown in Figure 2B, all emulsions exhibited a negative charge, which is consistent with the trend observed by Ye et al. (2018) for emulsions made from lipids and WPI, where the initial zeta potential also exhibited a negative charge. This is because the initial solution had a neutral pH (pH = 7), which is higher than the isoelectric points of whey protein (pI = 5.1) and casein (pI = 4.6), leading to a greater dissociation of acidic groups in the proteins, resulting in an overall negative charge (Zhang et al. 2015). In the system containing only MFGM emulsifier, the emulsion comprising coconut oil exhibited a significantly lower absolute value of droplet potential compared to the emulsions formed with the other two oil phases. This was attributed to the smaller droplet size of coconut oil, which contained medium‐chain fatty acids. Compared to the other two longer chain fatty acids, it resulted in a lower quantity of anionic polar lipids on the droplet surface (Bourlieu et al. 2016). Upon the addition of WPI and SCN, the surface charge density of droplets in all three oil systems notably decreased, due to the coverage of anionic polar lipids in the phospholipids on the fat globule surface by the added proteins, which exposes less negatively charged protein residues (Chen et al. 2020). This effect was particularly pronounced in the emulsion composed of coconut oil, because of its higher protein content on the droplet surface (Figure 2A).

#### 3.2.2 Particle Size and Distribution

**FIGURE 3:**
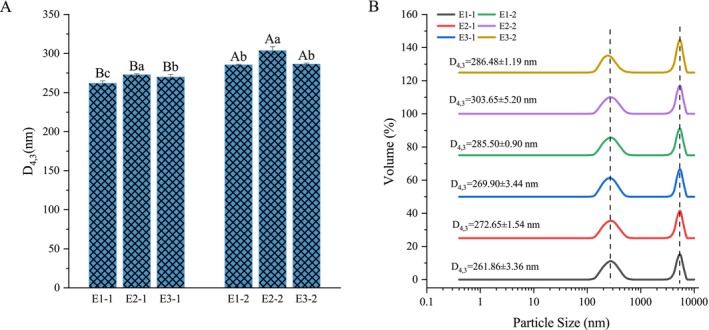
The particle size (A) and the size distribution (B) of six initial emulsions. In MFGM aqueous: E1‐1: Coconut oil; E2‐1: OPO; E3‐1: DHA algal oil. In mixed aqueous with MFGM, WPI, and SCN: E1‐2: Coconut oil; E2‐2: OPO; E3‐2: DHA algal oil. The different lowercase letters indicate significant differences between the same emulsifiers in different oil phases (*p* < 0.05), while uppercase letters indicate significant differences between the same oil phases in different emulsifiers (*p* < 0.05).

The particle size and the size distribution of the six emulsions would be affected by the carbon chain length of fatty acids in the emulsion and the protein content (Figure 3). In the emulsion system with only MFGM as the emulsifier, the D_4,3_ of OPO emulsion containing medium‐chain fatty acids was slightly higher than the other two emulsions. This result is consistent with the findings of Borel et al. (1996), which indicate a negative correlation between the carbon chain length of fatty acids and the median diameter of the emulsion. Moreover, the surface enrichment of different protein contents also influenced the size of fat droplets. This observation is in agreement with previously reported studies (Chen et al. 2020; Wang et al. 2017). Upon the addition of WPI and SCN to the emulsion, the D_4,3_ of all three emulsions significantly increased, with the particle size of OPO‐based droplets exhibiting a more pronounced increase compared to the other two emulsions. This indicated that the increase in protein content in the emulsifier system enhanced the influence of triglyceride carbon chain length on the D_4,3_ of fat droplets. However, neither of these two factors significantly affected the particle size distribution of the emulsion (Figure 3B). The distribution of emulsion particle sizes showed two peaks, occurring within the ranges of 112.4–615.1 nm and 3091–6439 nm.

#### 3.2.3 EAI and ESI

**FIGURE 4:**
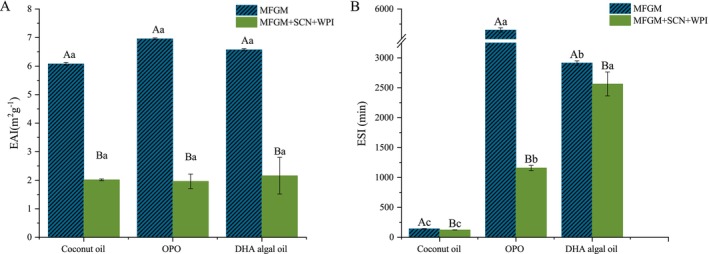
Effects of fatty acid carbon chain length and protein content in emulsions on emulsion activity index (EAI) (A) and emulsion stability index (ESI) (B). The different lowercase letters indicate significant differences between the same emulsifiers in different oil phases (*p* < 0.05), while uppercase letters indicate significant differences between the same oil phases in different emulsifiers (*p* < 0.05).

The emulsifying capabilities of proteins are quantified through the emulsion activity index (EAI) and the emulsion stability index (ESI). Although Michalski (2009) demonstrated that the emulsification capacity increases with the increase in the unsaturation of fatty acids and decreases with the elongation of the fatty acid chain, changes in the emulsifier content will also affect the emulsification performance of the emulsion (Figure 4).

In the absence of added WPI and SCN in the emulsion system, the values of EAI for emulsions composed of fatty acids of different chain lengths showed differences, yet not significant, whereas the differences in values of ESI were more pronounced (*p* < 0.05). The EAI and ESI values for the OPO emulsion were higher than those of the other two types of emulsions, indicating that the proteins adhered to the surface of OPO lipid droplets possess a higher emulsification capability and a stronger ability to prevent lipid droplet aggregation; emulsions formed by lipid droplets comprising longer chain fatty acids were more stable. Otherwise, lipid droplets formed by longer chain fatty acids displayed a higher charge density on their surface, thereby having an attribution to maintain the distance between droplets and preventing aggregation (Figure 2B). Upon increasing the protein content in the emulsifier, the EAI values of all three types of emulsions significantly decreased, with no significant differences between the groups. The stability index of emulsions formed by OPO lipids significantly decreased. This suggested that the addition of proteins diminished the impact of fatty acid carbon chain length on the emulsification capability of lipid droplets and reduced the emulsifying capacity of the emulsions. Although the addition of proteins increased the enrichment of proteins on the surface of lipid droplets, if their concentration exceeds that of the surfactants stabilizing the emulsion system, aggregation is highly likely to occur, thus destabilizing the original system (Zhang et al. 2022).

#### 3.2.4 The Storage Modulus and Loss Modulus

**FIGURE 5:**
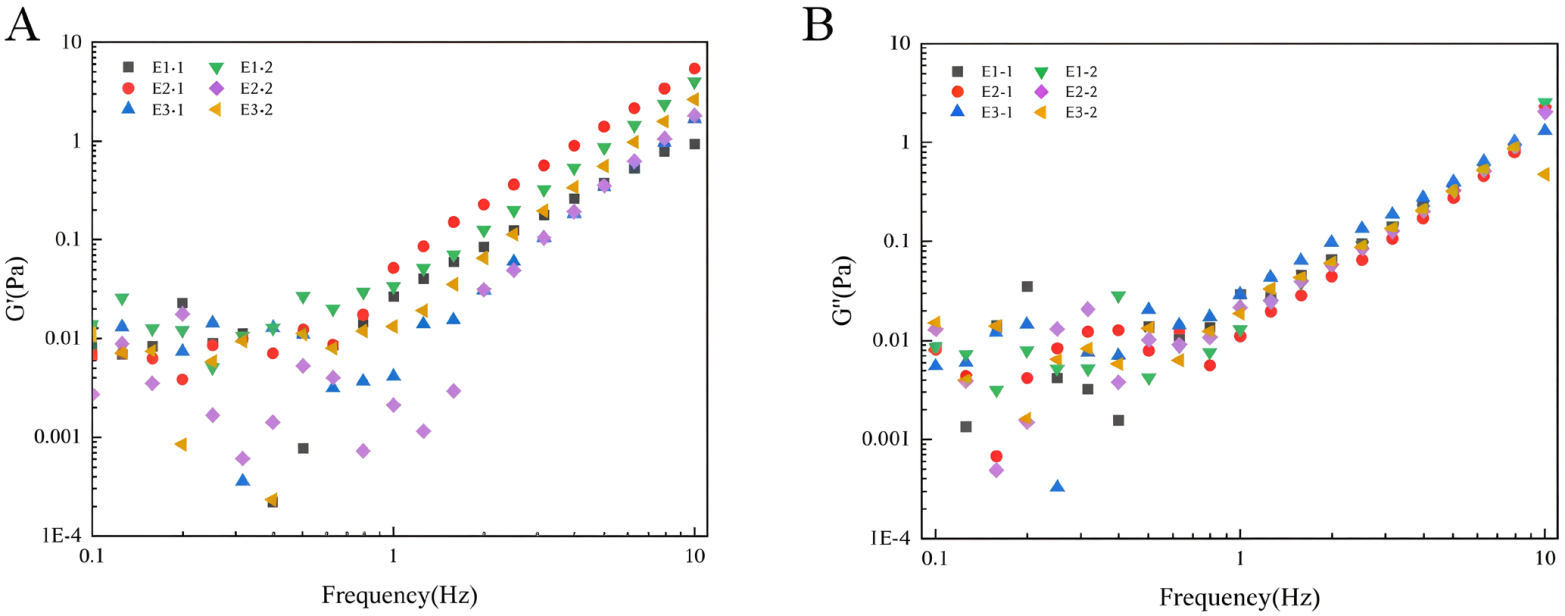
The frequency dependence of fatty acid carbon chain length and protein content on the storage modulus (G') (A) and loss modulus (G") (B) of emulsions.

The storage modulus (G'), also known as the elastic modulus, reflects the capacity of a material to store energy during cyclic deformation. It quantifies the strength of a material's elastic behavior, that is, how much deformation energy the material can store and recover upon stress removal. Materials with a higher G' exhibit stronger solid‐like characteristics or elasticity. The loss modulus (G"), also referred to as the viscous modulus, represents the capacity of a material to dissipate energy during cyclic deformation. This dissipated energy, released in the form of heat, is not recoverable for the material to return to its original shape. Materials with a higher G" display stronger fluid‐like properties or viscosity. When G' > G", it indicates that the emulsion can maintain its structure better under applied external stress, showing more elastic features; conversely, when G' < G", the emulsion flows more easily under applied stress, demonstrating more viscous characteristics. Therefore, the variation of G' and G" values with oscillation frequency provides important insights into the structural stability and rheological behavior of emulsions composed of fatty acids with different carbon chain lengths and varying protein content (Figure 5).

Within the 1–10 Hz range, emulsions exhibited a pronounced frequency dependence. All six emulsions demonstrated a consistent trend of increasing storage (G') and loss (G") moduli with the rise in an angular frequency range, with G' values marginally exceeding those of G". This suggested that emulsions formulated with proteins and MFGM as emulsifiers develop a structured, elastic gel network irrespective of the fatty acid chain length, exhibiting more pronounced elastic than viscous characteristics. Dimitrova and Leal‐Calderon (2004) also demonstrated that emulsions stabilized by certain proteins exhibit significantly higher elasticity. Notably, the difference in G' values, especially within emulsions solely stabilized by MFGM, was significant. This discrepancy was attributed to the influence of triglyceride chain length on the emulsification capacity of lipid droplets. The difference between G' and G" was significantly greater in emulsions comprising OPO, indicating a tighter droplet distribution within the MFGM and OPO‐based emulsions, and the structure of these emulsions was more stable. This finding was also corroborated in Figure 4B. Additional WPI and SCN into the emulsion system resulted in a reduced disparity between G' and G" values, suggesting that the increased proteins improved the viscosity of the emulsions and disrupted the previously stable and tight arrangement of components. The large surface coverage of droplets by proteins altered the original surface architecture of the droplets, weakened the repulsive forces between them, and enhanced the probability of droplet collisions and aggregation, thus impeding the relative mobility of droplets within the emulsion (Liu et al. 2023). These findings indicate that the impact of increased protein content as an emulsifier within the emulsion is much greater than the effect of the triglyceride chain length on droplet behavior.

### 3.3 In Vitro Digestion: The Hydrolysis of Fat Globules and Proteins in Six Emulsions

#### 3.3.1 The Rate of FFA Release

**FIGURE 6:**
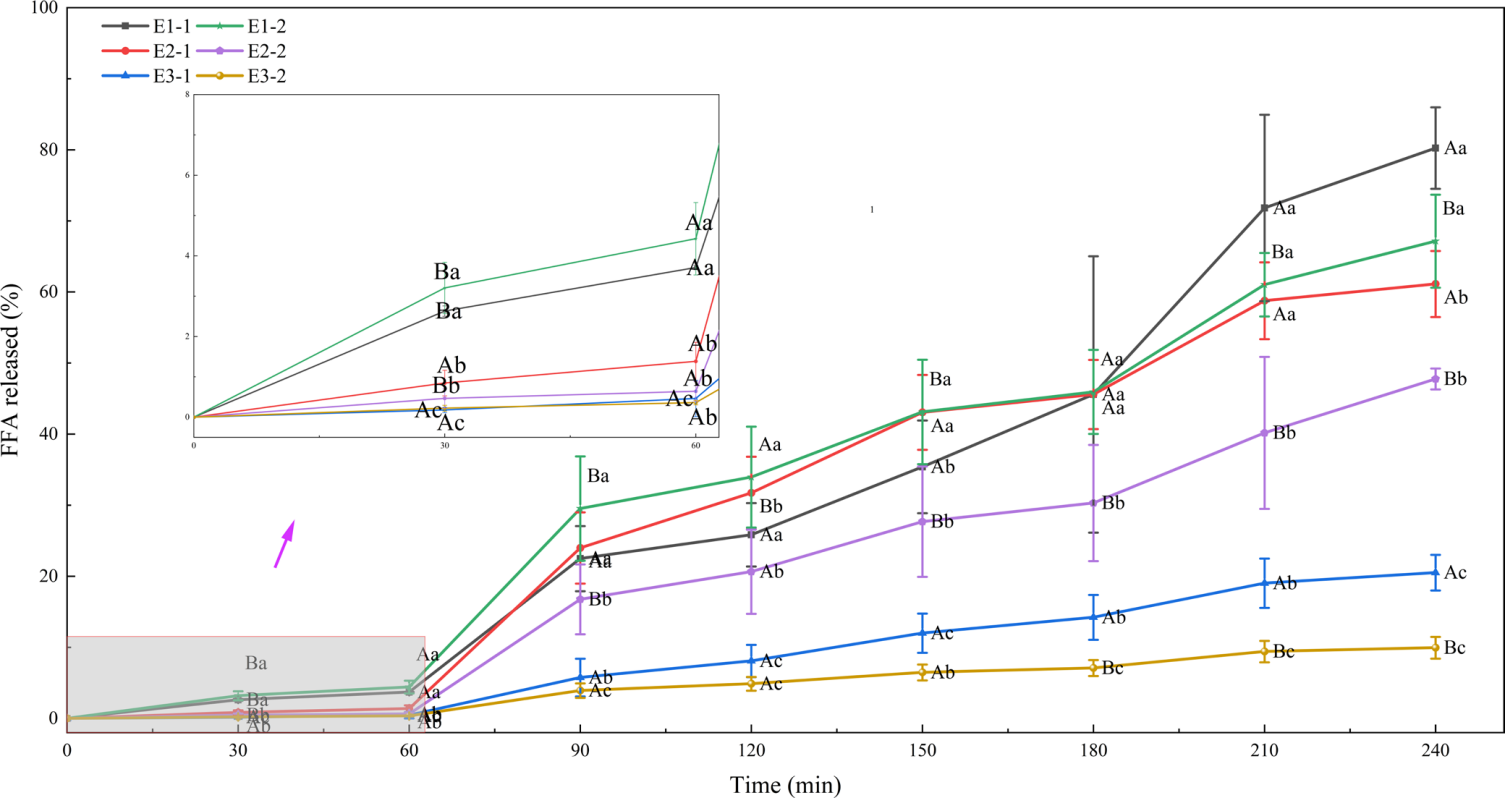
The FFA released degree of six emulsions in vitro gastrointestinal digestion. In MFGM aqueous: E1‐1: Coconut oil; E2‐1: OPO; E3‐1: DHA algal oil. In mixed aqueous with MFGM, WPI, and SCN: E1‐2: Coconut oil; E2‐2: OPO; E3‐2: DHA algal oil. The different lowercase letters indicate significant differences between the same emulsifiers in different oil phases (*p* < 0.05), while uppercase letters indicate significant differences between the same oil phases in different emulsifiers (*p* < 0.05).

In the in vitro simulated digestion system, the concentration of free fatty acids released at each time point reflects the extent of lipid hydrolysis (Figure 6). Throughout the digestion process, under the same emulsifier system, emulsions composed of coconut oil consistently exhibited significantly higher rates of fatty acid release compared to those formulated with the other two oils. This phenomenon was attributed to the smaller droplet size of triglycerides with shorter chain lengths, facilitating greater enzyme contact surface area. Furthermore, research by Dayrit (2015) indicates that the primary component of coconut oil, lauric acid, when ingested, is mostly transported directly to the liver, where it is rapidly converted into energy and other metabolites rather than being stored as fat. This explains the faster metabolism and easier absorption of coconut oil in the human body. Moreover, after fat globule breakdown, shorter chain fatty acids were more rapidly assimilated into bile salt micelles or vesicles, preventing accumulation on the surface of fat globules and impeding further enzyme–fatty globule interaction. This also explained why all six emulsions exhibited similar digestive trends in all six emulsions: a rapid increase in fatty acid release within the first 30 min of gastric digestion, followed by a slower increase; the same trend in the prior stages of intestinal digestion, followed by a slower increase, and a rapid increase in the last 60 min of lipolysis until reaching peak fat breakdown.

In systems with the same lipid composition but varying emulsifier concentrations, emulsions with lower protein content exhibit higher fatty acid release rates (Figure 6), which suggests that, in addition to the particle size of fat globules being a factor that affects the rate of fatty acid release, the surface structure of fat globules is also one of the influencing factors. This phenomenon was due to the increased presence of surface proteins on droplets, which reduced the contact area between fat enzymes and droplets. A similar result was reported by McClements and Li (2010), who demonstrated that a variety of surface‐active substances—such as endogenous compounds (e.g., proteins, phospholipids, and bile salts), exogenous substances (e.g., surfactants and proteins), and internally generated products (e.g., lipid digestion products)—present within the aqueous phase surrounding the lipid droplets compete with the surfactants at the oil–water interface. This competition alters the composition and properties of the fat droplet interface, thereby affecting the digestion of the droplets. Moreover, the elevated levels of casein could lead to the formation of aggregates with proteases in the digestive system, creating a condensed network structure that encases droplets and slows down fatty acid release (Ma et al. 2024). Particularly in the gastric digestive system, the isoelectric point of casein was near the pH maintained during gastric digestion. The aggregation of proteins disrupts the interactions between droplets in the original emulsion, particularly pronounced in systems with higher protein content, where the electrostatic repulsion between lipid droplets with lower surface charge is more likely to be destroyed (Liang et al. 2018).

#### 3.3.2 Condition of Proteolysis

**FIGURE 7:**
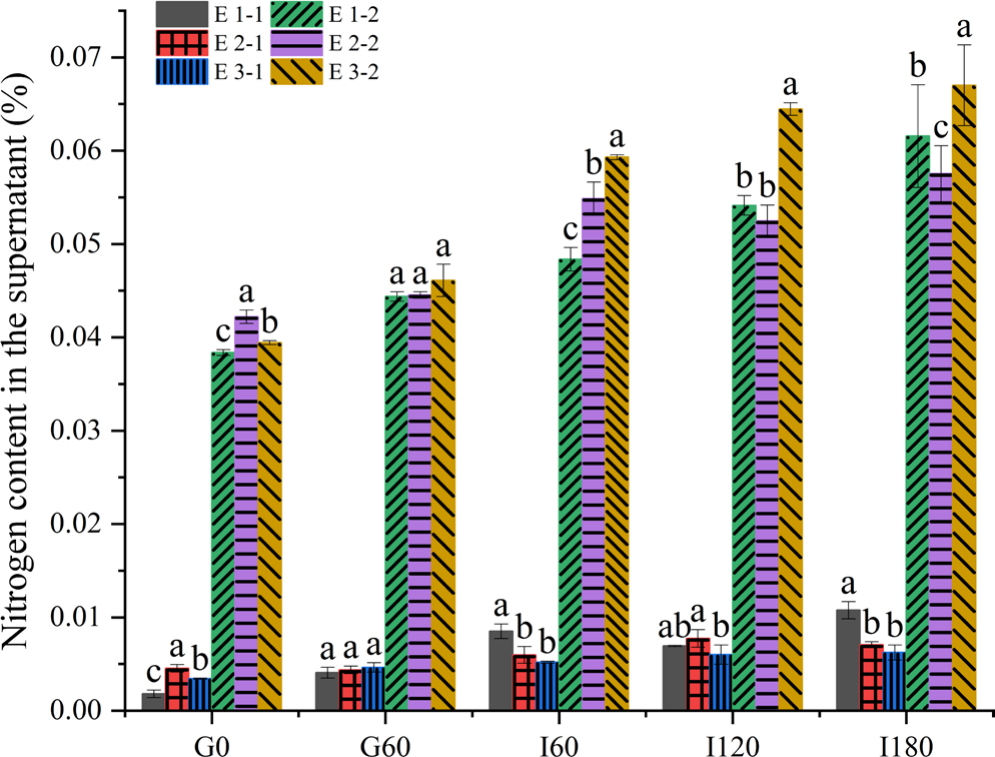
Free amino acid released the condition of six emulsions in vitro gastrointestinal digestion. E1‐1: Coconut oil in MFGM aqueous. E2‐1: OPO in MFGM aqueous. E3‐1: DHA algal oil in MFGM aqueous. E1‐2: Coconut oil in mixed aqueous with MFGM, WPI, and SCN. E2‐2: OPO in mixed aqueous with MFGM, WPI, and SCN. E3‐2: DHA algal oil in mixed aqueous with MFGM, WPI, and SCN. The different lowercase letters indicate significant differences between emulsions composed of the same aqueous phase with three different oil phases (*p* < 0.05).

The digestibility of proteins serves as a fundamental criterion for evaluating nutritional quality, indirectly reflecting the efficiency of protein utilization and the extent of protein hydrolysis into amino acids for absorption (Corzo‐Martínez et al. 2012). Based on the data presented in Figure 7, following gastric digestion, the three oils exhibited no significant impact on the extent of protein digestion. However, during the intestinal digestion phase, significant differences in protein digestion were observed among emulsions composed of different oil types. This suggests a notable alteration in the structural properties of substances during the intestinal digestion phase among the six emulsions, consequently influencing protein digestion. The increase in emulsifier content alters the trend of protein digestion, attributable to the contact area between proteins and proteases during emulsion digestion. This is consistent with the findings of Zhu et al. (2021), who investigated the impact of phospholipid and protein content at the oil–water interface on protein digestibility. This may be attributed to the presence of phospholipids, bile salts, or free fatty acids, which reduce the zeta potential of fat droplets in the water‐in‐oil emulsions stabilized by protein (Malaki et al. 2011; Zhang et al. 2015). In the MFGM system, emulsions composed of coconut oil exhibited higher protein digestibility, while in the whey protein‐casein system, emulsions composed of DHA algae oil released higher levels of amino acids. This observation may be attributed to differences in the stability of emulsions formed by coconut oil and DHA algae oil (Figure 4). The higher stability of emulsions formed by DHA algae oil is conducive to reducing protein aggregation in the emulsion system.

## 4 Conclusion

The varying carbon chain lengths of fatty acids significantly impact the physicochemical properties and digestion of emulsions, particularly in terms of emulsification capacity at the droplet interface and fatty acid release rates. The addition of proteins significantly reduces the differences in the influence of triglyceride carbon chain length on droplet interface emulsification capability, breaks down the interdroplet interaction forces, and markedly enhances the differences in droplet surface charge density and the rate of fatty acid release during early intestinal digestion. This may offer a novel approach or insight for formulating droplets using singular oil mixed, contributing to enhancing the bioavailability of proteins and fats in emulsions. Additionally, it may contribute to the development of food products tailored for populations with compromised digestive absorption capacities, such as infant/premature infant formulas or lipid‐functional foods for the elderly/special populations. Future research endeavors will further explore the impact of these two factors on the bioavailability of lipids in emulsions from the perspective of nutrient absorption.

## Author Contributions

**Yanchen Liu:** data curation (lead), methodology (lead), validation (lead), writing – original draft (lead), writing – review and editing (lead). **Ning Wang:** methodology (supporting). **Yunpeng Xu:** investigation (supporting), software (supporting). **Zihao Guo:** methodology (supporting), writing – review and editing (supporting). **Guangqing Mu:** funding acquisition (lead), resources (equal), supervision (equal). **Xuemei Zhu:** funding acquisition (lead), resources (equal), supervision (lead), writing – review and editing (supporting).

## Conflicts of Interest

The authors declare no conflicts of interest.

## Data Availability

The data that support the findings of this study are available from the corresponding author upon reasonable request.
